# Risk of collagen-related disorders and neurological events among patients with uncomplicated urinary tract infection following short treatment with fluoroquinolones: a cohort study

**DOI:** 10.1128/aac.00690-24

**Published:** 2024-10-29

**Authors:** Fanny S. Mitrani-Gold, Shinyoung Ju, Myriam Drysdale, Anna Schultze, George Mu, John Logie

**Affiliations:** 1 GSK, Collegeville, Pennsylvania, USA; 2 GSK, London, United Kingdom; 3 London School of Hygiene and Tropical Medicine, London, United Kingdom; Johns Hopkins University School of Medicine, Baltimore, Maryland, USA

**Keywords:** adverse events of special interest, collagen events, neurological events, fluoroquinolones, standard-of-care, antibiotics, uncomplicated urinary tract infection, risk

## Abstract

Studies of fluoroquinolone (FQ) safety across indications show increased collagen/neurological adverse event (AE) risk, yet patients still receive FQs for uncomplicated urinary tract infections (uUTIs). This retrospective, cohort study investigated the risk of collagen/neurological AEs of special interest (AESIs) with short-term FQ use versus standard-of-care antibiotics (trimethoprim–sulfamethoxazole [SXT], nitrofurantoin [NTF]) among female outpatients with uUTIs. This study was conducted between December 2009 and 2019 using Optum’s de-identified Clinformatics Data Mart Database. Adjusted absolute risks were calculated for composite/collagen/neurological AESIs (Kaplan–Meier cumulative hazards, after applying stabilized inverse probability of treatment weighting [sIPTW]). Adjusted hazard ratios were generated (sIPTW Cox proportional hazard modeling). Overall, 954,777 patients were included: FQ (*n* = 386,537 [40.5%]); SXT (*n* = 237,120 [24.8%]); NTF (*n* = 314,585 [32.9%]). Adjusted absolute risk range for collagen/neurological AESIs was <1%–4.5%. The hazard (95% CI) of tendon rupture was 25% higher with FQ versus SXT (1.25 [1.00–1.57]; *P* = 0.0497). Patients receiving FQ had lower hazard of neurological (0.95 [0.93–0.97]; *P* < 0.0001), central nervous system (0.85 [0.80–0.89]; *P* < 0.0001), and peripheral nervous system (0.96 [0.93–0.98]; *P* = 0.0016) AESIs versus NTF. Following a short treatment duration, FQs were associated with increased risk of tendon rupture versus SXT and reduced risk (adjusted hazard ratios) of neurological AESI versus NTF. Individual patient risk and consequences for known uncommon, yet serious, AEs need to inform appropriate antibiotic choice in treating uUTIs. Patient profile, efficacy, microbiome impact, safety, and surveillance should inform antibiotic selection for uUTI management, in accordance with guidelines.

## INTRODUCTION

Uncomplicated urinary tract infections (uUTIs) are among the most common outpatient bacterial infections in women in the United States (US) (1, 2), with an estimated annual incidence of 11% (2). More than half of all adult women (50%–60%) will have at least one uUTI in their lifetime (2). Urinary tract infections (UTIs) may have frequent recurrences and potential complications, and are a significant cause of morbidity (1). The majority of UTIs acquired in the community among female patients are uncomplicated, meaning they are not associated with structural or functional abnormalities of the urinary tract, or comorbidities, such as complicated or uncontrolled diabetes, immunosuppression, or pregnancy (3).

Treatment of community-acquired uUTIs remains largely empiric in the US, and other treatment guidelines (where available) recommend first-line treatment with trimethoprim–sulfamethoxazole (SXT), nitrofurantoin (NTF), or fosfomycin (4–6). The optimal choice of agent for the treatment of uUTIs depends on several factors and should be made on an individual patient basis (5). Although SXT, NTF, and fosfomycin are recommended first-line treatments in the US (4), a significant number of patients receive alternate antibiotics as empiric therapy (7). Fluoroquinolones (FQs) are broad-spectrum antimicrobials (8) that are widely prescribed to treat uUTIs (9). However, the US Food and Drug Administration (FDA) has issued “black box” warnings for FQ agents due to their association with disabling and potentially long-lasting adverse events (AEs) affecting tendons, muscles, joints, and the central nervous system (CNS) (7, 10). The Infectious Diseases Society of America recommends that FQs are used only in patients who have no other UTI treatment options (5), and guidance from the European Medicines Agency states that FQs should be avoided in patients who have previously had serious side effects with a FQ antibiotic, and used with special caution in the elderly, patients with kidney disease, and those who have had an organ transplantation; combined use of FQ and systemic corticosteroids should also be avoided (11, 12).

Despite an overall decline in FQ prescribing following the FDA warnings in 2013 and 2016, many US patients with uUTIs continue to be treated empirically with FQs (13). Although there is some evidence of an increase in the risk of collagen AEs (most notably tendonitis and tendon rupture) (14), and neurological AEs with FQ use (15), prior studies of AE risk have looked at cross-indication populations with variation in the use of drugs included within the FQ class, treatment durations, and disease severities (14, 16–20). It remains unclear whether any increased risk of collagen and neurological AEs is observed with short-term use of FQs. For this reason, and to reduce heterogeneity, it is important to understand the potential AE risk associated with the short-term use of oral FQs specifically for uUTI treatment. Therefore, this study sought to investigate the association between short-term use of FQs and AEs of special interest ([AESIs], collagen and neurological, independently) in patients with uUTI, to estimate the risk of these AESIs in patients treated with FQ versus standard-of-care antibiotics for uUTIs (SXT, NTF, and amoxicillin clavulanate [AMC; added *post hoc* to contextualize FQ-related AESIs]).

## MATERIALS AND METHODS

### Study design and participants

**Fig 1:**
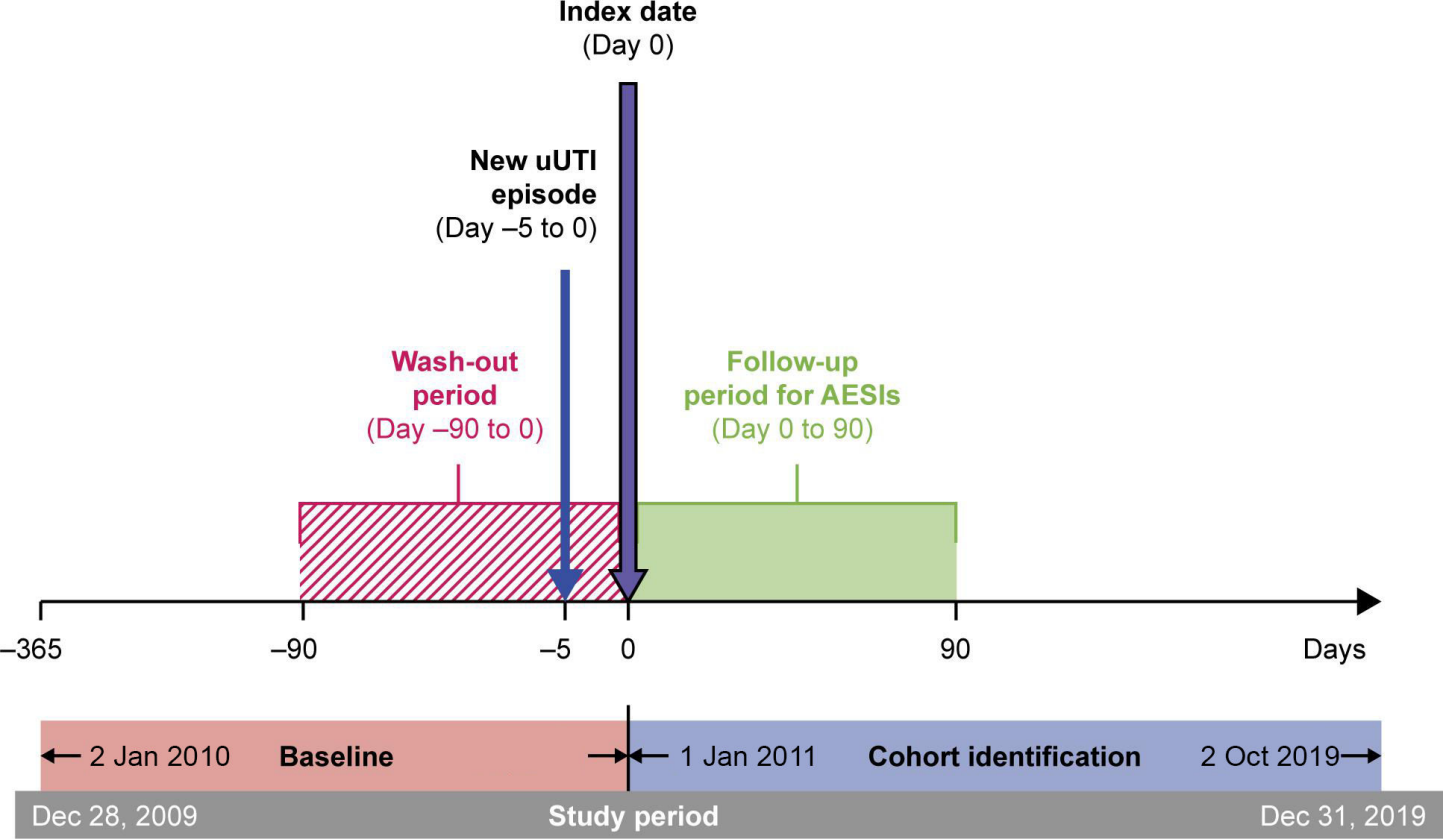
Study design. AESIs, adverse events of special interest; uUTI, uncomplicated urinary tract infection.

This was a retrospective comparative cohort study that used patient data from Optum’s de-identified Clinformatics Data Mart Database, a large database of commercial and Medicare Advantage health claims incorporating members from all 50 US states. Patients with a new uUTI, defined by the presence of an International Classification of Diseases, Ninth and Tenth Revisions, Clinical Modification (ICD-9/10 CM) diagnosis code for UTI following ≥28 days after a “no prior UTI” diagnosis, were identified between 1 January 2011 and 2 October 2019. The date of antibiotic prescription for the uUTI episode was considered the “index” date (Day 0). The study design (Fig. 1) comprised a 12-month pre-index baseline period to collect patient history data, and up to 90-days of follow-up to assess AESIs. To prevent misclassification of antibiotic exposure, patients were censored based on subsequent antibiotic prescriptions or changes in therapy; hence, some patients were followed for <90 days. A 90-day pre-index window with no prior exposure was implemented to remove any residual effects from prior antibiotic exposures to the treatments being evaluated (including intravenous [IV] formulations for FQs and SXT). All patients were required to have ≥365 days of continuous health plan enrollment pre-index. No restrictions were applied on post-index follow-up.

Eligible patients were female aged ≥12 years who had received outpatient treatment for a new uUTI episode with a 3–10-day prescription of one of the following oral antimicrobials within 5 days of the uUTI episode (determined at the time of issue, and not by quantifying actual exposure): FQ (ciprofloxacin, levofloxacin, norfloxacin, or ofloxacin), SXT, NTF, or AMC. Patients were excluded if they had a prescription of longer than 10 days or had received >1 oral antimicrobial (concurrently) at the index date. Patients who did not meet all inclusion and exclusion criteria at prescription date were eligible for inclusion at a later date (if the subsequent uUTI event was treated within 5 days, where all inclusion and exclusion criteria were met). Diagnostic codes indicative of acute cystitis (with or without hematuria), cystitis (unspecified), or UTI (site not specified) were used to identify uUTI cases, and codes are provided in the supplementary material (Table S1).

Patients were excluded if they were considered to have a complicated UTI (cUTI) based on the following criteria: UTI episode was in an inpatient setting; IV antibiotic use within 5 days of the UTI diagnosis; structural or functional abnormalities of the urinary tract or urological procedures associated with cUTI in the year prior to an UTI episode; complicated or uncontrolled diabetes mellitus in the year prior; immunosuppression or treatment with immunosuppressive therapy in the year prior; pregnancy in the prior 9 months plus 28 days; or human immunodeficiency virus (HIV) any time prior to UTI diagnosis. Patients were also excluded if they had any collagen or neurological AESIs in the 90 days prior to index (to identify treatment-related AEs following treatment), or genetic syndromes or autoimmune diseases potentially associated with the measured AESIs in the prior 365 days (Table S1). Patients with controlled or uncomplicated diabetes mellitus were eligible for inclusion if all other inclusion/exclusion criteria were met.

In the context of this study, AESIs were collagen or neurological events identified via ICD-9/10 CM diagnostic codes using claims data (Table S1); however, the AESIs in this study have not undergone a medical record evaluation/adjudication.

Eligible patients were censored at the earliest of the following: additional antibiotic prescription (FQ, SXT, NTF, AMC; oral or IV); date of death (from different sources contributing to mortality database); first break in continuous enrollment; or 90 days post-index. This was performed for both absolute risk and hazard ratio estimations.

### Variables

The primary analysis of the study assessed the absolute risk (%) and hazard ratios of collagen and neurological AESIs for each antibiotic class during the 90-day follow-up period; hazard ratios were used to compare time-to-event for FQ versus standard-of-care antibiotic treatments. Risk definitions are detailed in Table S2.

The following collagen AESIs were evaluated in the FQ versus SXT cohorts: composite collagen AEs (any of the following collagen AEs), tendon rupture, retinal detachment, uveitis, aortic aneurysm, aortic dissection, and aortic aneurysm with dissection (AA/AD). The following neurological AESIs were evaluated in the FQ versus NTF cohorts: composite neurological AEs (any of the following neurological AEs), CNS AEs (seizures/convulsions, intracranial hypertension, psychosis/delirium, and altered mental status/encephalopathy), and peripheral nervous system (PNS) AEs (muscle weakness, paresthesia/sensory disturbances, gait dysfunction, and peripheral neuropathy).

The comparators differed for collagen and neurological AESIs, as described below, and were selected based on the published literature. For collagen AESIs, SXT was the comparator because it is a first-line antibiotic for uUTI (5, 21); we did not select NTF as a comparator for the assessment of collagen AESI, given the manufacturer labeling recommendation to avoid NTF in patients with creatine clearance of <60 mL/min (22). Our rationale was that collagen AESIs are more prevalent among the elderly, and the use of NTF as the comparator may have not allowed for the assessment of increased risk due to age, if there was not sufficient NTF use in elderly females with uUTI (23). Nonetheless, we recognize that there is recent evidence supporting the use of NTF in patients with susceptible uropathogens and a creatinine clearance of 30–60 mL/min (23, 24). For neurological AESIs, NTF was the comparator because SXT is known to have effects on the nervous system (25). AMC was initially considered as a comparator in the study; however, it is used as an alternate antibiotic for the treatment of uUTI in patients with known allergies or intolerance to first-line treatment, and we selected active comparators that were first-line and commonly used. AMC was added as a comparator *post hoc*, after viewing the initial study results, the methods and results are included in the supplementary materials. Fosfomycin was not considered as an active comparator in the study because it is used in <3% of female patients with uUTIs in the US (26).

The 12-month baseline (pre-index) period was used to describe patient characteristics and to assess covariates that could confound the association between exposure and the AESI. The following demographics were assessed at index: age (continuous and categorical); geographical region; index year; race/ethnicity. The following clinical characteristics and measures of prior healthcare utilization were assessed during the baseline period: recurrent uUTIs (one additional uUTI episode in the prior 6 months or two additional episodes in the prior 12 months); prior antimicrobial exposure (Day −91 to −184 and Day −185 to −364); ≥1 all-cause hospitalization; ≥1 all-cause physician visit; and ≥1 prior UTI (cUTI and uUTI). Comorbidities assessed in the prior 12 months were based on the Charleston Comorbidity Index (CCI) (27), and comedications of interest in the 90 days pre-index were also assessed (details in the supplementary materials).

### Statistical analysis

Demographics, prior healthcare utilization, comorbidities, and comedications identified during baseline were analyzed using descriptive statistics. Mean and standard deviation (SD) were reported for continuous variables and number and percentages for categorical variables. Crude absolute risks with 95% confidence intervals (CIs) were calculated via Kaplan–Meier cumulative hazards for composite (collagen and neurological) AESIs and for each type of collagen or neurological AESI. Kaplan–Meier survival curves were generated for composite AESIs. The log-rank test was used (*P* < 0.05 was significant) to evaluate the overall difference in the Kaplan–Meier curves.

Propensity scores were derived from the predicted probability of receiving FQ treatment, estimated in a multivariable logistic regression model with FQ treatment versus standard-of-care treatments (SXT and NTF) fitted as a binary outcome. *A priori* covariates included demographics/clinical (age [continuous], race/ethnicity, region, index year, recurrence of an uUTI, and prior antimicrobial exposure), healthcare utilization (prior hospitalization and prior physician visits), prior UTI, comorbidities, and comedications. Stabilized inverse probability of treatment weighting (sIPTW) was used to balance baseline covariates by using generated propensity scores to weight each individual with FQ exposure by the inverse probability of having the FQ exposure multiplied by the proportion of patients with the FQ exposure in the population, while each individual with the comparator exposures (SXT or NTF) was weighted by the inverse (or reciprocal) of one minus the probability of having comparator exposures (SXT or NTF) multiplied by the proportion of patients with the comparator exposure (SXT or NTF) in the population.

Covariate balance was assessed prior to and after sIPTW, with standardized differences >10% interpreted as showing a significant imbalance between FQ and a standard-of-care treatment (SXT or NTF). Adjusted absolute risks (95% CI) were calculated via cumulative hazards through the Kaplan–Meier method after the application of sIPTW (28).

Crude hazard ratios of collagen or neurological AESIs between FQ and standard-of-care cohorts were first generated; sIPTW-weighted Cox proportional hazard models were used to produce adjusted hazard ratios with *P* < 0.05 considered statistically significant. Patients were censored as described above for calculation of absolute risks. The potential impact of unmeasured confounders was quantified via E-values, and the proportional hazard (PH) assumptions were tested via Schoenfeld Residuals. In the event that the overall Cox proportional hazard model for an outcome violated the PH assumption (*P* < 0.05), then period-specific Cox-adjusted hazard ratios would have been generated (Days 1–30, 31–60, 61–90) by fitting a time by exposure interaction term, allowing adjusted hazard ratios to vary over time.

All statistical analyses were performed using SAS studio 3.81 (SAS Institute, Cary NC, USA) (29).

## RESULTS

### Descriptive data

**Fig 2:**
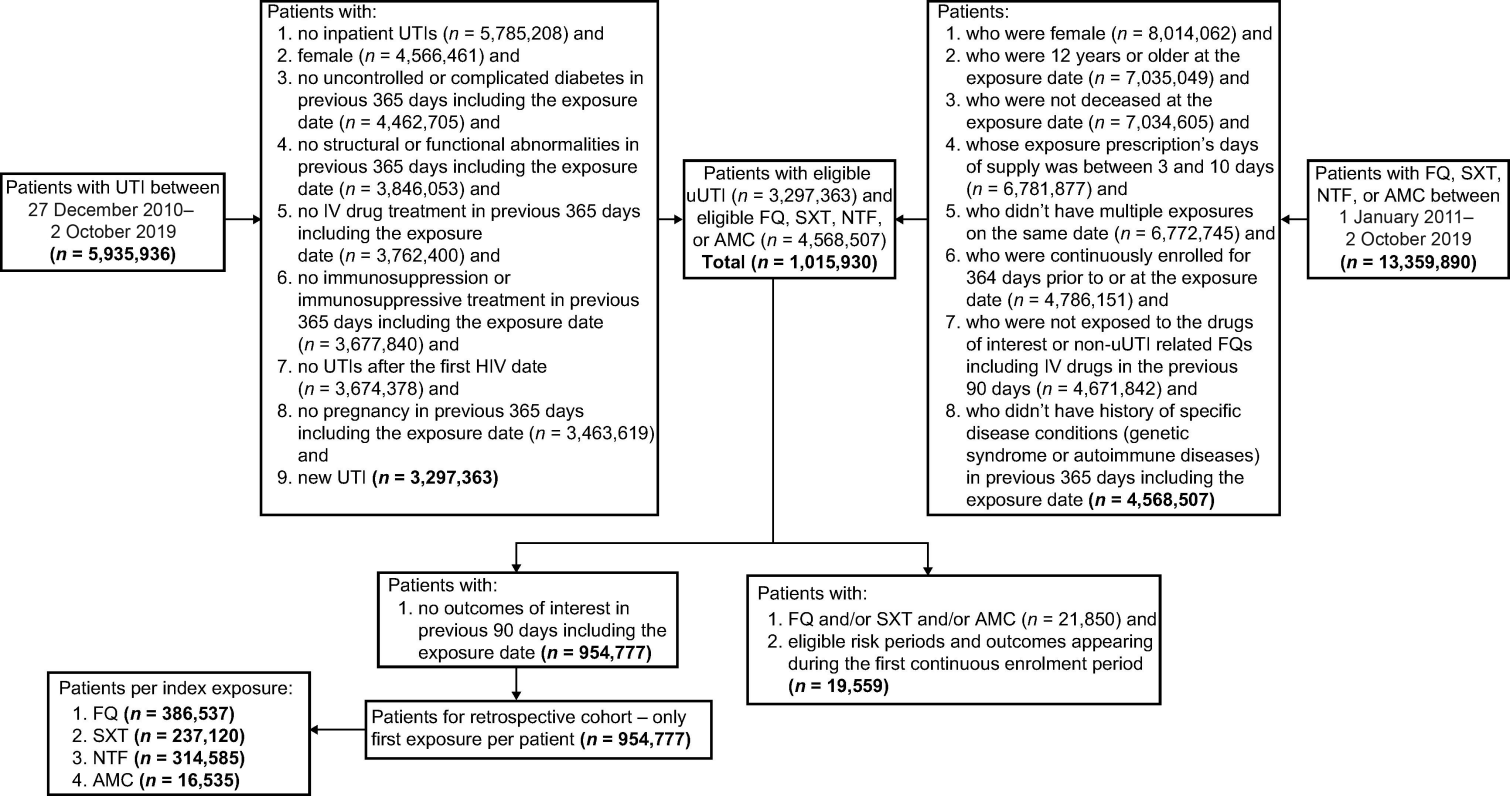
Patient attrition diagram. AMC, amoxicillin clavulanate; FQ, fluoroquinolone; HIV, human immunodeficiency virus; IV, intravenous; NTF, nitrofurantoin; SXT, trimethoprim/sulfamethoxazole; uUTI, uncomplicated urinary tract infection; UTI, urinary tract infection.

Overall, 954,777 female US outpatients were included in the study, of whom 386,537 (40.5%) received FQ, 237,120 (24.8%) received SXT, and 314,585 (32.9%) received NTF (Fig. 2; Table 1). The overall mean age (SD) at index was 50.3 (±20.1) years; patients in the FQ cohort were older than those in the SXT and NTF cohorts (mean, 54.0 years versus 47.9 and 47.4 years, respectively). The cohorts were similar with regard to race and ethnicity. The number of patients with recurrent uUTI, defined as one previous uUTI episode in the prior 6 months or two episodes in the prior 12 months, was 29,507 (7.6%) in the FQ group, 14,719 (6.2%) in the SXT group, and 21,115 (6.7%) in the NTF group (Table 1). In the 12 months before index, patients in the FQ cohort had a higher proportion of antimicrobial exposure, all-cause hospitalization, and UTI recurrence compared with the SXT and NTF cohorts (Table 1). In the FQ, SXT, and NTF cohorts, chronic obstructive pulmonary disease was observed in 1.7%, 1.3%, and 0.9% of patients, respectively, and uncomplicated or controlled diabetes was observed in 1.4%, 1.2%, and 1.1% of patients, respectively (Table 1).

**TABLE 1:** Baseline patient characteristics per index antibiotic treatment group (FQ versus SXT versus NTF)

	Index antibiotic treatment groups			
Baseline characteristics	Overall (*N* = 954,777)	FQ (*N* = 386,537)	SXT (*N* = 237,120)	NTF (*N* = 314,585)
Mean age at index (years ± SD)	50.3 ± 20.1	54.0 ± 19.3	47.9 ± 20.9	47.4 ± 19.6
Race, n (%)				
White	651,557 (68.2)	261,108 (67.6)	164,720 (69.5)	215,201 (68.4)
Black	88,895 (9.3)	36,386 (9.4)	23,884 (10.1)	27,012 (8.6)
Asian	34,897 (3.7)	14,614 (3.8)	7,255 (3.1)	12,436 (4.0)
Hispanic	115,822 (12.1)	49,234 (12.7)	26,176 (11.0)	37,919 (12.1)
Unknown	63,606 (6.7)	25,195 (6.5)	15,085 (6.4)	22,017 (7.0)
Treatment in prior 12 months, n (%)				
Antimicrobial exposure^*a*^	496,382 (52.0)	207,138 (53.6)	120,409 (50.8)	159,064 (50.6)
All-cause hospitalization	49,535 (5.2)	22,830 (5.9)	11,271 (4.8)	13,864 (4.4)
Physician visits	944,531 (98.9)	382,281 (98.9)	234,539 (98.9)	311,524 (99.0)
≥1 UTI episode	154,969 (16.2)	67,644 (17.5)	34,763 (14.7)	48,600 (15.4)
Recurrent uUTI, n (%)				
One episode in prior 6 months or 2 in prior 12	67,270 (7.0)	29,507 (7.6)	14,719 (6.2)	21,115 (6.7)
One episode in prior 6 months	58,949 (6.2)	25,829 (6.7)	12,924 (5.5)	18,495 (5.9)
Two episodes in prior 12 months	27,402 (2.9)	12,103 (3.1)	5,832 (2.5)	8,570 (2.7)
Drug use in prior 90 days, n (%)				
Diabetes drug(s)	48,232 (5.1)	21,860 (5.7)	11,420 (4.8)	13,842 (4.4)
Corticosteroids	49,289 (5.2)	21,160 (5.5)	11,548 (4.9)	15,068 (4.8)
Aminoglycosides	18 (0.0)	7 (0.0)	5 (0.0)	6 (0.0)
Tetracyclines	8,220 (0.9)	3,086 (0.8)	1,853 (0.8)	3,089 (1.0)
Statins	141,257 (14.8)	69,087 (17.9)	32,311 (13.6)	36,962 (11.7)
Gabapentinoids	4,408 (0.5)	2,004 (0.5)	1,018 (0.4)	1,276 (0.4)
Isoniazid	0	0	0	0
Comorbidities, n (%)				
Total	954,777 (100.0)	386,537 (100.0)	237,120 (100.0)	314,585 (100)
Myocardial infarction	754 (<1)	384 (<1)	165 (<1)	182 (<1)
Congestive heart failure	2,273 (<1)	1,205 (<1)	454 (<1)	507 (<1)
Peripheral vascular disease	2,286 (<1)	1,254 (<1)	494 (<1)	456 (<1)
Cerebrocardiovascular disease	1,918 (<1)	961 (<1)	416 (<1)	487 (<1)
Dementia	3,610 (<1)	1,893 (<1)	795 (<1)	837 (<1)
Chronic obstructive pulmonary disease	13,376 (1.4)	6,754 (1.7)	3,129 (1.3)	2,854 (0.9)
Connective tissue/rheumatic disease	1,060 (<1)	585 (<1)	213 (<1)	230 (<1)
Peptic ulcer disease	281 (<1)	150 (<1)	62 (<1)	63 (<1)
Mild liver disease	1,877 (<1)	1,010 (<1)	364 (<1)	451 (<1)
Diabetes, uncomplicated/controlled	12,308 (1.3)	5,592 (1.4)	2,923 (1.2)	3,371 (1.1)
Renal disease mild to moderate	938 (<1)	566 (<1)	184 (<1)	156 (<1)
Paraplegia and hemiplegia	165 (<1)	81 (<1)	39 (<1)	40 (<1)
Cancer	3,028 (<1)	1,662 (<1)	670 (<1)	615 (<1)
Severe liver disease	59 (<1)	29 (<1)	13 (<1)	11 (<1)
Metastatic carcinoma	244 (<1)	155 (<1)	47 (<1)	37 (<1)

^*a*^ Antimicrobial exposure 91–364 days prior to the index date. Patient data were obtained from Optum’s de-identified Clinformatics Data Mart Database. FQ, fluoroquinolone; NTF, nitrofurantoin; SD, standard deviation; SXT, trimethoprim/sulfamethoxazole; uUTI, uncomplicated urinary tract infection; UTI, urinary tract infection.

Collagen AESIs within 90 days of treatment without the application of censoring were observed in 1,200 patients (0.31%) treated with FQ and 564 patients (0.24%) treated with SXT (Table 2). Neurological AESIs were observed in 18,899 patients (4.89%) in the FQ cohort and 12,111 patients (3.85%) in the NTF cohort (Table 2).

**TABLE 2:** Descriptive number (%) of collagen and neurological AESIs within 90 days of follow-up by index antibiotic treatment (without the application of Kaplan–Meier method)

	Index antibiotic treatment	
Collagen AESIs, n (%)^*a*^	FQ (*N* = 386,537)	SXT (*N* = 237,120)
Composite	1,200 (0.31)	564 (0.24)
Tendon rupture	308 (0.08)	115 (0.05)
Retinal detachment	173 (0.05)	96 (0.04)
Uveitis	262 (0.07)	139 (0.06)
Aortic aneurysm	444 (0.11)	209 (0.09)
Aortic dissection	25 (0.01)	14 (0.01)

Neurological AESIs, n (%)^*a*^	FQ (*N* = 386,537)	NTF (*N* = 314,585)
Composite	18,899 (4.89)	12,111 (3.85)
CNS	3,722 (0.96)	2,295 (0.73)
PNS	16,420 (4.25)	10,565 (3.36)

^*a*^ Some patients experienced both CNS and PNS AEs. AESIs, adverse events of special interest; CNS, central nervous system; PNS, peripheral nervous system; FQ, fluoroquinolone; NTF, nitrofurantoin; SXT, trimethoprim–sulfamethoxazole.

### Absolute risks of collagen and neurological AESIs

**Fig 3:**
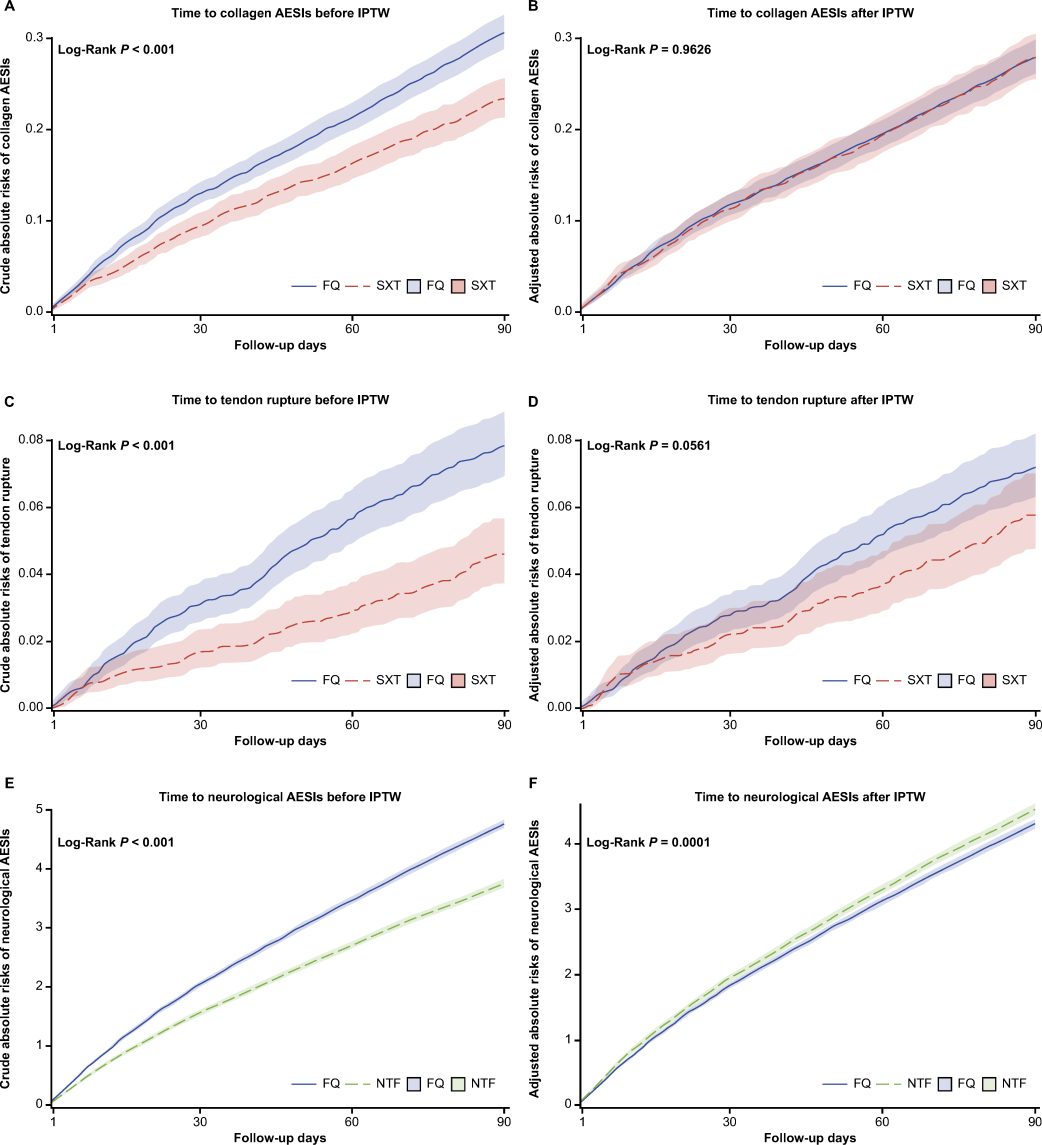
Crude and adjusted absolute risks over time by index antibiotic treatment: (**A and B**) collagen AESIs (aggregate), (**C and D**) tendon rupture, and (**E and F**) neurological AESIs (aggregate). AESI, adverse event of special interest; FQ, fluoroquinolone; NTF, nitrofurantoin; sIPTW, inverse probability of treatment weighting; SXT, trimethoprim/sulfamethoxazole.

The crude absolute 90-day risk of collagen AESIs in the FQ and SXT groups was 0.31% (95% CI, 0.29–0.33) and 0.23% (95% CI, 0.21–0.26), respectively (Table 3). The crude absolute risk of collagen AESIs was higher for FQ versus SXT (log rank *P* < 0.001; Fig. 3A), but there was no difference after covariate adjustment (log rank *P* = 0.9626; Fig. 3B). Similarly, there was a higher crude absolute risk of tendon rupture over time between FQ and SXT (log rank *P* < 0.001; Fig. 3C), but this was attenuated after covariate adjustment (log rank *P* = 0.0561; Fig. 3D).

**TABLE 3:** Crude absolute risk of collagen and neurological AESIs by index antibiotic treatment (with censoring)

Collagen and neurological AESIs	Index antibiotic treatment crude absolute risk, % (95% CI)^*a*^	
	FQ (*N* = 386,537)	SXT (*N* = 237,120)
Any collagen AESIs	0.31 (0.29–0.33)	0.23 (0.21–0.26)
Tendon rupture	0.08 (0.07–0.09)	0.05 (0.04–0.06)
Retinal detachment	0.05 (0.04–0.05)	0.04 (0.03–0.05)
Uveitis	0.07 (0.06–0.08)	0.06 (0.05–0.08)
Aortic aneurysm	0.11 (0.10–0.12)	0.08 (0.07–0.10)
Aortic dissection	0.01 (0.00–0.01)	0.01 (0.00–0.01)

	FQ (*N* = 386,537)	NTF (*N* = 314,585)
Any neurological AESIs	4.76 (4.69–4.84)	3.75 (3.68–3.83)
CNS	0.90 (0.87–0.93)	0.70 (0.67–0.73)
PNS	4.14 (4.07–4.21)	3.27 (3.20–3.34)

^*a*^ Assessed from Days 1–90. AESIs, adverse events of special interest; CI, confidence interval; CNS, central nervous system; PNS, peripheral nervous system; FQ, fluoroquinolone; NTF, nitrofurantoin; SXT, trimethoprim–sulfamethoxazole.

The crude absolute 90-day risk of neurological AESIs was higher for FQ versus NTF (FQ: 4.76% [95% CI, 4.69–4.84] versus NTF: 3.75% [95% CI, 3.68–3.83], log rank *P* < 0.001; Table 3; Fig. 3E) before censoring and remained so after covariate adjustment (log rank *P* = 0.0001; Fig. 3F). In both the FQ and NTF groups, PNS AESIs were approximately four times more frequent than CNS AESIs (Table 3).

**Fig 4:**
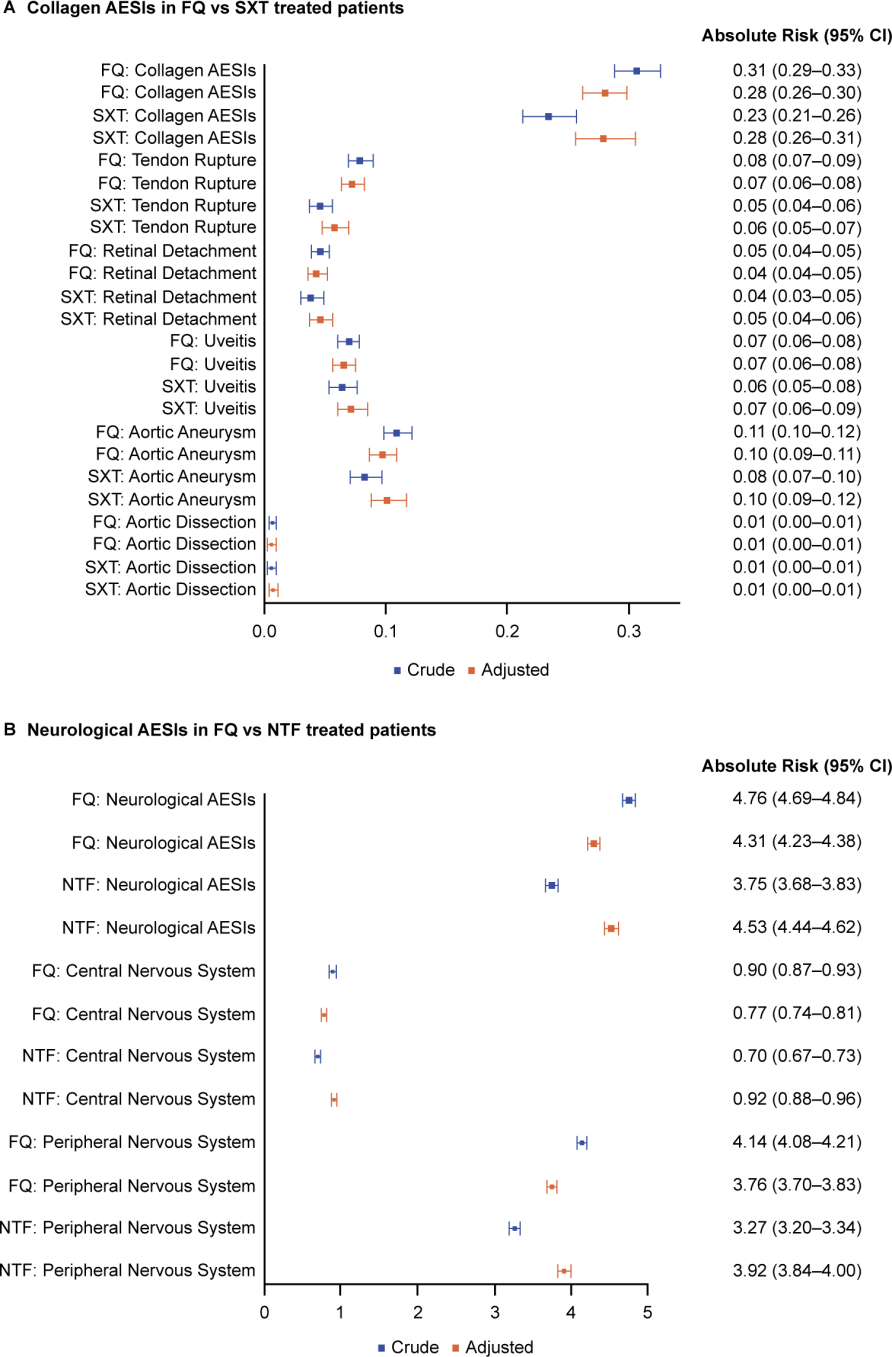
Forest plot of crude and adjusted absolute risks of (**A**) collagen and (**B**) neurological AESIs. Collagen AESIs comprised tendon rupture, aortic aneurysm with or without dissection, retinal detachment, uveitis, and a composite category for all collagen AESIs. Neurological AESIs comprised CNS AESIs (seizures/convulsions, intracranial hypertension, psychosis/delirium, and altered mental status/encephalopathy), PNS AESIs (muscle weakness, paresthesia/sensory disturbance [tingling, numbness, burning pain, and allodynia], gait dysfunction, and peripheral neuropathy), and a composite category for all CNS and PNS AESIs. AESIs, adverse events of special interest; CNS, central nervous system; FQ, fluoroquinolone; NTF, nitrofurantoin; PNS, peripheral nervous system; sIPTW, inverse probability of treatment weighting; SXT, trimethoprim/sulfamethoxazole.

After covariate adjustment, the absolute risk of collagen AESIs (composite and individual) was <1%; this was comparable in the FQ and SXT cohorts (Table 4; Fig. 4A). There was a numerically higher adjusted absolute risk of tendon rupture in the FQ cohort versus the SXT cohort (0.07% versus 0.06%), but this was not statistically significant (log rank *P* = 0.0561). For neurological AESIs, the adjusted absolute risk was significantly lower in the FQ cohort compared with the NTF cohort for composite neurological AESIs (4.31% versus 4.53%), CNS AESIs (0.77% versus 0.92%), and PNS AESIs (3.76% versus 3.92%) (Table 4; Fig. 4B).

**TABLE 4:** Adjusted absolute risk of collagen and neurological AESIs by index antibiotic treatment (after sIPTW)

Collagen and neurological AESIs	Index antibiotic treatment adjusted absolute risk, % (95% CI)^*a*^	
	FQ (*N* = 386,537)	SXT (*N* = 237,120)
Any collagen AESIs	0.28 (0.26–0.30)	0.28 (0.26–0.31)
Tendon rupture	0.07 (0.06–0.08)	0.06 (0.05–0.07)
Retinal detachment	0.04 (0.04–0.05)	0.05 (0.04–0.06)
Uveitis	0.07 (0.06–0.07)	0.07 (0.06–0.08)
Aortic aneurysm	0.10 (0.09–0.11)	0.10 (0.09–0.12)
Aortic dissection	0.01 (0.00–0.01)	0.01 (0.00–0.01)

	FQ (*N* = 386,537)	NTF (*N* = 314,585)
Any neurological AESIs	4.31 (4.23–4.38)	4.53 (4.44–4.61)
CNS	0.77 (0.74–0.81)	0.92 (0.88–0.96)
PNS	3.76 (3.70–3.83)	3.92 (3.84–4.00)

^*a*^ Assessed from Days 1–90. AESIs, adverse events of special interest; CI, confidence interval; CNS, central nervous system; FQ, fluoroquinolone; sIPTW, inverse probability of treatment weighting; NTF, nitrofurantoin; PNS, peripheral nervous system; SXT, trimethoprim/sulfamethoxazole.

### Hazard ratios of collagen and neurological AESIs

**Fig 5:**
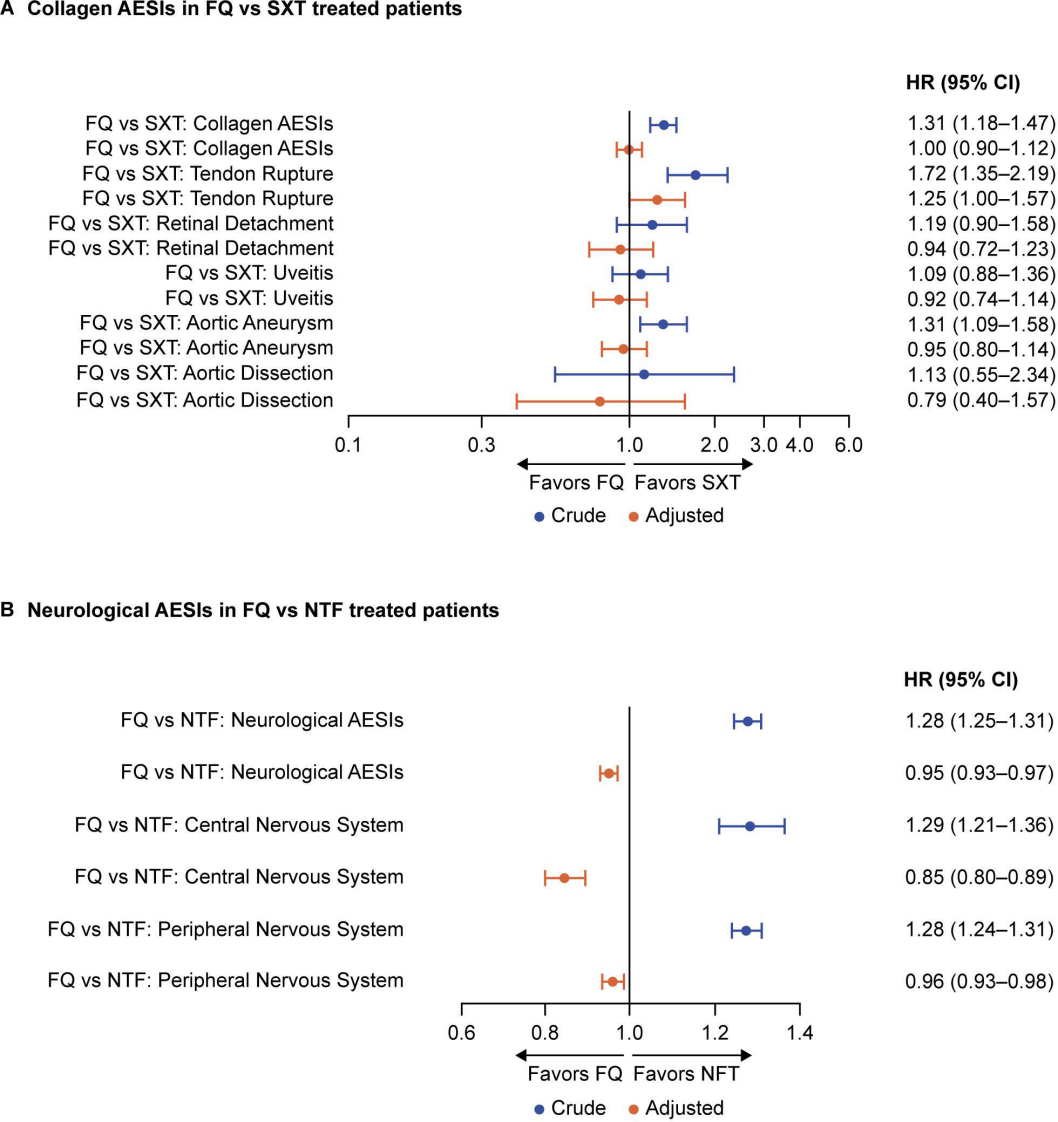
Forest plot of crude and adjusted hazard ratios for (**A**) collagen and (**B**) neurological AESIs. Collagen and neurological AEs are aggregate. Reference HR = 1. HRs are adjusted for age, race/ethnicity, region, year of new uUTI, uUTI recurrence, prior antimicrobial exposure, prior hospitalization, prior physician visits, prior UTI, comorbidities, and comedications. AESIs, adverse events of special interest; CI, confidence interval; FQ, fluoroquinolone; HR, hazard ratio; NTF, nitrofurantoin; PNS, peripheral nervous system; sIPTW, inverse probability of treatment weighting; SXT, trimethoprim/sulfamethoxazole.

Before adjusting for patient characteristics, the FQ cohort had a 31% higher crude hazard for composite collagen AESIs (1.31; 95% CI, 1.18–1.47), a 72% higher crude hazard for tendon rupture (1.72; 95% CI, 1.35–2.19), and a 31% higher crude hazard for aortic aneurysm (1.31; 95% CI, 1.09–1.58), versus the SXT cohort (Fig. 5A). There were no treatment differences for the other collagen AESIs. Compared with the NTF cohort, patients in the FQ cohort had a 28% higher crude hazard for composite neurological AESIs (1.28; 95% CI, 1.25–1.31), with a 29% higher crude hazard for CNS AESIs (1.29; 95% CI, 1.21–1.36), and a 28% higher crude hazard for PNS AESIs (1.28; 95% CI 1.24–1.31) (Fig. 5B).

After covariate adjustment, the higher crude hazard for AESIs seen in the FQ cohort versus standard-of-care cohorts were all attenuated to either no added risk or a decreased risk, except for tendon rupture (Fig. 5). For collagen AESIs, the only AE for which a significant treatment difference remained after adjustment was tendon rupture, with the FQ cohort having a 25% higher adjusted hazard compared with the SXT cohort within 90 days (1.25; 95% CI, 1.00–1.57; *P* = 0.0497) (Table 5; Fig. 5A). For composite and all other collagen AESIs, adjusted hazard ratios of ≤1 were observed with 95% CIs that crossed one (Table 5; Fig. 5A). Compared with the NTF cohort, patients in the FQ cohort had a 5% lower adjusted hazard for composite neurological AESIs (0.95; 95% CI, 0.93–0.97; *P* < 0.0001), a 15% lower adjusted hazard for CNS AESIs (0.85; 95% CI, 0.78–0.89; *P* < 0.0001), and a 4% lower adjusted hazard for PNS AESIs (0.96; 95% CI, 0.93–0.98; *P* = 0.0016) (Table 5; Fig. 5B). None of the Cox models for collagen or neurological AESIs violated the PH assumption (Table 5).

**TABLE 5:** Adjusted hazard ratio of collagen and neurological AESIs by index antibiotic treatment (after sIPTW)

Collagen and neurological AESI	Adjusted hazard ratio (95% CI)	*P*-value	E-values	PH assumption *P*-value^*a*^
Collagen AESIs: FQ versus SXT				
Any collagen AE	1.00 (0.90–1.12)	0.9615	1.0539	0.7690
Tendon rupture	1.25 (1.00–1.57)	0.0497	1.8185	0.5887
Retinal detachment	0.94 (0.72–1.23)	0.6378	1.3335	0.9984
Uveitis	0.92 (0.74–1.14)	0.4270	1.4068	0.6517
Aortic aneurysm	0.95 (0.80–1.14)	0.6050	1.2714	0.8297
Aortic dissection	0.79 (0.40–1.57)	0.4992	1.8500	0.7748
Neurological AESIs: FQ versus NTF				
Any neurological AE	0.95 (0.93–0.97)	<0.0001	1.2941	0.2664
CNS	0.85 (0.80–0.89)	<0.0001	1.6490	0.9046
PNS	0.96 (0.93–0.98)	0.0016	1.2555	0.3603

^*a*^ Assessed from Days 1–90; *P* < 0.05 is considered as the violation of PH assumption. AESIs, adverse events of special interest; CI, confidence interval; CNS, central nervous system; FQ, fluoroquinolone; NTF, nitrofurantoin; PH, proportional hazards; PNS, peripheral nervous system; sIPTW, inverse probability of treatment weighting; SXT, trimethoprim/sulfamethoxazole.

### E-values

In the Cox models, the E-values for individual AESIs ranged from 1.26 to 1.85 and were all larger in magnitude than each of the observed adjusted hazard ratios for collagen and neurological AESIs when comparing FQ with SXT and NTF, respectively (Table 5). For the observed adjusted hazard ratios of 0.96 for PNS AESIs and 1.25 for tendon rupture, an unmeasured confounder with an adjusted hazard ratio of ≥1.25 and ≥1.82, respectively, would be required to explain the observed associations in the study. All non-composite AESI E-values were larger than the Cox-adjusted hazard ratios observed, which suggests that a significant level of unmeasured confounding would be needed to explain away the hazard ratios observed in the study.

### *Post hoc* analyses (AMC)

After reviewing initial study results, an AMC comparator group was added *post hoc* for both collagen and neurological AESIs, since both SXT and NTF comparators had a potential known association with various AESIs (23, 25). The AMC cohort included 16,535 (1.7%) uUTI patients who were compared with 386,537 patients treated with FQ (Table S3). The AMC cohort was much smaller than the other treatment cohorts, and the sample size was determined to not be statistically powered for comparisons, after running the primary analyses. The methods and results for the AMC group are fully described in the supplementary material.

## DISCUSSION

The current study describes the short-term risk of collagen and neurological AESIs among female patients with uUTI, treated with FQ versus different standard-of-care antibiotics. Our results reported an association between FQ treatment and increased risk of tendon rupture when compared with SXT (after adjustment for patient characteristics) in the uUTI population, despite treatment durations being relatively short (and limited to FQs used for uUTI). In addition, the results of our study (based on adjusted hazard ratios) suggested that exposure to FQs versus SXT was not associated with an increased risk for retinal detachment, uveitis, aortic aneurysm, or aortic dissection. There was no significant association between FQ use and increased risk for CNS- or PNS-related AESIs; however, there was a significantly lower risk of neurological AESIs in patients receiving FQ compared with NTF.

### FQ and tendon rupture

Despite the statistically significant association between FQ and tendon rupture observed in the current study, the magnitude of the increase in risk (25% higher adjusted hazard) was at the lower end of values observed in the literature (30–32). Kim *et al*. studied musculoskeletal AEs among pediatric patients in Korea treated with FQ compared with AMC and found a slightly increased risk of musculoskeletal AEs after FQ treatment (propensity score-matched cohort: adjusted hazard ratio, 1.19; 95% CI, 1.01–1.40; *P* = 0.042) (31), which was similar to the risk reported in the current study. In another study, Daneman *et al*. concluded that, compared with non-users, current FQ use (across indication) was associated with an increased risk of tendon rupture (adjusted hazard ratio, 2.40; 95% CI, 2.24–2.57) and aortic aneurysm (adjusted hazard ratio, 2.24; 95% CI, 2.02–2.49) (30), which was almost twice the reported magnitude of increase in risk between FQ and tendon rupture compared with the current study. Similarly, a meta-analysis reported that FQ treatment, compared with non-users and/or comparator antibiotics, was associated with significant (*P* < 0.001) increase in the odds of any tendon disorder (nine studies, one FQ versus comparator antibiotic; odds ratio, 1.98; 95% CI, 1.62–2.43), Achilles tendonitis (three studies, one FQ versus comparator antibiotic; odds ratio, 3.95; 95% CI, 3.11–5.01), and Achilles rupture (eight studies, one FQ versus comparator antibiotic; odds ratio, 2.52; 95% CI, 1.81–3.52) (32).

Our study included a large cohort from across the US and robust statistical methodology to account for a large number of measured potential demographic and clinical confounders. Potential differences in design that distinguish our study from previous work, such as type(s) and severity of indication and treatment duration, may explain why the increased risk of tendon rupture with FQ exposure we observed was lower than in previous studies. To mitigate the potential issue of unobserved differences between cohort exposure groups, we included a homogeneous population of adult and adolescent females with a single indication, a “new” episode of uUTI treated with an oral antibiotic (FQ, SXT, NTF, and AMC) in the outpatient setting for a relatively short treatment duration (3–10 days). The inclusion of a 90-day pre-index exposure washout window limited prior antimicrobial exposures that could lead to misclassification and associated bias.

### FQ and aortic aneurysm and aortic dissection

There is conflicting evidence describing the association between FQ use and aortic aneurysm and aortic dissection. Using a case-crossover design across indications, Lee *et al.* reported increased odds of exposure to FQ during the 60 days prior to AA/AD versus the reference period (odds ratio, 2.71; 95% CI, 1.14–6.46) (33). Pasternak *et al.* observed an increased risk of AA/AD with FQs versus AMC (hazard ratio, 1.66; 95% CI, 1.12–2.46) across indications using a propensity score-matched cohort (34). In a nested case–control study by Dong *et al.*, the odds ratio (95% CI) of AA/AD comparing indicated infections with no indicated infection, adjusted for concomitant antibiotic use, was 1.73 (1.66–1.81) (35). However, FQ was not associated with an increased odds of AA/AD compared with AMC or ampicillin–sulbactam. In a population-based nationwide cohort study, Chen *et al.* found that the risk of AA/AD in patients with any UTI was not significantly different between use of FQs and first or second generation cephalosporins (adjusted hazard ratio, 0.86 [95% CI, 0.59–1.27]) (16); the absence of an association may in part have been driven by higher mortality in the FQ group. A recent meta-analysis of 22 observational studies (across indications) on the risk of collagen-related events associated with FQ exposure versus no exposure has also concluded that FQ use was associated with aortic dissection and aortic aneurysm (36).

### Other collagen AESIs

Existing literature on the associations between FQ treatment and uveitis and retinal detachment are also inconsistent (19, 20, 36–51). Inconsistencies between studies that assessed the risk of collagen AEs post FQ exposure are thought to be due to a combination of confounding by indication, conflation of effects by patient use of multiple antibiotics in a short period of time, lack of adjustment for covariates, and poor balance of baseline characteristics between comparators (even after adjustment). Some previous studies did not adjust for clinically relevant covariates and/or consider the impact of potential antibiotic re-exposures (due to uUTIs or other indications requiring FQs) between the index exposure and AEs (37, 38, 50). In contrast, our study provides evidence on the risk of AESIs with FQ compared with other antibiotics used in uUTI treatment, after addressing potential biases, exposure misclassification, and confounding by indication.

### FQ and neurological AESIs

Our study did not find an association between FQ use and increased risk of CNS- or PNS-related AESIs compared with standard-of-care; rather, we observed significantly lower adjusted risks of any neurological CNS- and PNS-related AESIs in patients who received FQ compared with NTF. The association between FQ use and neurological events is not well characterized in the literature. To date, one epidemiological study has examined the association between FQ exposure and CNS dysfunction, and two studies have examined the association between FQ exposure and PNS dysfunction. Ellis *et al.* conducted a propensity score-matched prospective cohort study using claims data to assess the association between FQ exposure and nervous system disturbances, relative to therapeutic alternatives, among patients with acute bacterial sinusitis, acute bacterial exacerbation of chronic bronchitis, uUTI, or acute bronchitis (15). Compared with the current study, Ellis *et al.* reported a numerically higher risk of CNS (8%) and PNS (9%) dysfunction associated with FQ exposure relative to therapeutic alternatives; the hazard ratio (95% CI) for CNS and PNS dysfunction associated with FQ exposure was 1.08 (1.05–1.11) and 1.09 (1.07–1.11), respectively (15). Morales *et al.* observed an adjusted incident rate ratio (95% CI) for peripheral neuropathy of 1.47 (1.13–1.92) with FQ use versus no FQ exposure (AMC), the risk increasing with additional days of exposure (51). Similarly, Etminan *et al.* reported that current users of FQ had a higher relative risk of peripheral neuropathy (1.83; 95% CI, 1.49–2.27) compared with nonusers of FQ (52). An explanation for the lower risk of neurological AESIs with FQ use (versus NTF) in our cohort of patients with uUTI may be due to inclusion of FQs not indicated for uUTI in prior studies investigating AESIs across varied indications, exposures, severities, and duration.

### Limitations

While our study population comprised patients from Optum’s de-identified Clinformatics Data Mart Database, which incorporates members from all 50 US states, it may nevertheless not be fully representative of uUTI as the database is limited to patients (and dependents) insured through their employers or with a Medicare Advantage plan. Moreover, potential heterogeneity that may exist at the regional level due to differences in local guidelines is not captured. Our results may not be generalizable to other countries, where patient demographics and treatment patterns may differ considerably. Some FQs known to be more strongly associated with AEs, such as moxifloxacin, were not included in our study as they are not used to treat uUTIs. Exposure measurement was based on antibiotic prescriptions dispensed, and patients were censored according to subsequent antibiotic prescriptions or changes in therapy to prevent misclassification of exposure. While antibiotics dispensed were captured accurately in the claims database, treatment adherence could not be measured in the study (it was assumed that, if dispensed, antibiotics were taken as prescribed). Studies using claims databases offer advantages for studying rare outcomes but may lack important clinical information, such as laboratory and microbiology results and symptoms, which are important for uUTI diagnosis, and severity at enrollment. In the cases where date of death was not available in Optum’s de-identified Clinformatics Data Mart Database, follow-up times might not have been accurately censored. To account for variation in the duration of FQ treatment across various real-world practice settings (primary care, obstetrics and gynaecology, urology, urgent care, etc.), female patients with uUTI who were treated with FQs for a duration of 3–10 days were eligible for the study after meeting all inclusion/exclusion criteria. Given that the 2011 Infectious Diseases Society of America guideline (5) recommend 3 days of FQ as an alternative treatment for uUTI, and that our study captured some patients with uUTI with longer FQ treatment based on real-world prescribing practices across providers, our results may overestimate the risk of AESIs due to a 3-day course of FQ treatment. Allowing only 3 days of FQ treatment as the study inclusion criteria would only include patients with uUTI who were strictly treated according to guidelines and not aligned with real-world prescribing practices. The exclusion of patients with concomitant use of antibiotics for uUTI in our study was implemented to reduce misclassification bias; however, this may have contributed to the decreased observed risk (adjusted hazard ratios) when compared with other studies. Patients with hemiplegia and paraplegia (captured based on ICD-9/10 codes) may have permanent or temporary catheters and which could be misclassified as uUTI if the urine source (catheter) was not specified for the index urine specimen; however, this represented <1% of the study population.

### Conclusions

Compared with first-line antibiotics (SXT and NTF) approved for the treatment of uUTIs in the US, FQs were associated with an increased risk (based on adjusted hazard ratios) of tendon rupture but not with an increased risk of other collagen (when compared with SXT) or neurological (when compared with NTF) AESIs. This study provides further evidence informing physicians on the risk of collagen and neurological AESIs with short-term FQ treatment versus standard-of-care antibiotics (SXT and NTF) specifically among female adolescent and adult patients with uUTIs. Individual patient risk and consequences for known uncommon, yet serious AESIs need to inform appropriate antibiotic choice in the management of uUTI. Efficacy, impact on the microbiome, safety, and surveillance should all inform antibiotic selection, in accordance with guidelines.

## Data Availability

The data that support the findings of this study were made available to the authors through third-party license from Optum’s de-identified Clinformatics Data Mart Database (Clinformatics), a commercial data provider in the United States. As such, the authors cannot make these data publicly available due to a data use agreement. Other researchers can access these data by purchasing a license through Clinformatics. Inclusion criteria specified in Materials and Methods would allow other researchers to identify the same cohort of patients used for these analyses. Interested parties may see https://www.optum.com/business/life-sciences/real-world-data/claims-data.html for more information on Clinformatics.

## SUPPLEMENTAL MATERIAL

Supplemental material aac.00690-24-s0001.docx Supplemental methods and results, Tables S1 to S8, and Fig. S1 to S3.

ASM does not own the copyrights to Supplemental Material that may be linked to, or accessed through, an article. The authors have granted ASM a non-exclusive, world-wide license to publish the Supplemental Material files. Please contact the corresponding author directly for reuse.
