# Supplementary material for: Risk of collagen-related disorders and neurological events among patients with uncomplicated urinary tract infection following short treatment with fluoroquinolones: a cohort study
Source: Antimicrob Agents Chemother. 2024 Oct 29;68(12):e00690-24. doi: 10.1128/aac.00690-24 (PMC11619313; doi:10.1128/aac.00690-24)
Supplement: Supplemental material — Supplemental methods and results, Tables S1 to S8, and Fig. S1 to S3. [file aac.00690-24-s0001.docx]

**Supplementary materials**

**ADDITIONAL METHODS**

***Post hoc* analysis**

After reviewing initial study results, both comparators for the primary analysis (trimethoprim-sulfamethoxazole [SXT] and nitrofurantoin [NTF]) had potential associations with a number of study adverse events of special interest (AESIs), making it difficult to contextualize the AESIs related to fluoroquinolones (FQs). To help further understand the risks associated with FQs, an amoxicillin clavulanate (AMC) comparator group was added. AMC was initially considered as a comparator in the study but was not selected as it is recommended as an alternative treatment for uncomplicated urinary tract infections (uUTI) rather than empirical or first-line therapy.

Methods and statistical analysis for the *post hoc* analyses were consistent with the primary analyses of this study (see ‘Methods’ and ‘Statistical analysis’ sections in the main manuscript).

***Duration of index antibiotic treatment***

*Post hoc* analysis of the dataset was performed using descriptive statistics to calculate the mean, standard deviation (SD) and median (range [min, max]) duration of treatment for each of the treatment groups.

**ADDITIONAL RESULTS**

***Post hoc* analysis**

Overall, 954,777 female outpatients were included in the study, of whom 386,537 (40.5%) received FQ and 16,535 (1.7%) received AMC (Table S3). The number of patients in the AMC group was small and the statistical power was insufficient for comparisons between FQ and AMC cohorts and should be interpreted with caution. Baseline patient characteristics are shown in Table S3. Patients in the FQ and AMC cohorts were comparable in age (mean age 54.0 years versus 54.5 years, respectively). The AMC cohort had a slightly higher proportion of Hispanic patients and a slightly lower proportion of white patients (compared to the FQ cohort).

Patients in the AMC cohort had a greater recent healthcare burden in the 12 months before index, with a higher proportion of patients with antimicrobial exposure (59.1% versus 53.6%), hospitalization (9.5% versus 5.9%), and UTI recurrence (11.7% versus 7.6%) compared with the FQ cohort (Table S3).

The reported numbers of collagen and neurological AESIs for each treatment cohort during follow-up are shown in Table S4. Composite collagen AESIs within 90 days were observed in 1,200 of 386,537 patients (0.31%) treated with FQ compared with 73 of 16,535 patients (0.44%) treated with AMC. Composite neurological AESIs occurred in 18,899 of 386,537 patients (4.89%) in the FQ cohort compared with 1,000 of 16,535 patients (6.05%) treated with AMC.

**Absolute risk of AESIs**

Overall, the crude absolute risk of collagen AESIs was very low in the FQ and AMC groups, and the crude absolute risk of neurological AESIs was greater than for collagen AESIs in both cohorts, over the 90-day follow-up (Table S5). The crude absolute risk of AESIs affecting the peripheral nervous system (PNS) compared with the central nervous system (CNS) was approximately 4-fold higher in the FQ cohort and 3-fold higher in the AMC cohort. Before weighting, there was a trend towards increased cumulative composite collagen AESIs over time in the AMC cohort versus the FQ cohort. However, the difference was non-significant (log rank *P* = 0.0779) (Figure S1A). The unadjusted cumulative incidence of composite neurological AESIs over time was higher for AMC versus FQ (log rank *P* < 0.001) (Figure S1C).

After covariate adjustment, the absolute risks of collagen AESIs (composite and individual) were very low (<1%) in the FQ and AMC treatment cohorts (Table S6). There were no significant differences in the adjusted absolute risk or time-to-event of any collagen AESIs when comparing FQ with AMC (Figure S2A). However, adjusted absolute risks of composite neurological AESIs, CNS AESIs, and PNS AESIs were all higher in the AMC cohort compared with the FQ cohort, with no overlap in confidence intervals (Figure S2B; Table S6).

**Hazard ratios of AESIs**

For the 90-day follow-up period, there were no significant between-treatment differences in the crude hazard ratio of composite collagen AESIs, tendon rupture, uveitis, aortic aneurysm, and aortic dissection among FQ- versus AMC-treated patients, with wide 95% confidence intervals (CIs) that crossed 1 (Table S7; Figure S3A). However, the adjusted hazard for retinal detachment was 49% lower for FQ compared with AMC (0.51, 95% CI 0.28–0.92) (Table S7; Figure S3A).

For the 90-day follow-up period, the FQ cohort had a 16% lower adjusted hazard for composite neurological AESIs (0.84, 95% CI 0.78–0.90; *P* < 0.0001), which was 30% lower for CNS AESIs (0.70, 95% CI 0.60–0.81; *P* < 0.0001), and 13% lower for PNS AESIs (0.87, 95% CI 0.80–0.94; *P* = 0.0007) when compared with the AMC cohort (Table S7; Figure S3B). There was no evidence that any of the models for collagen or neurological AESIs violated the proportional hazards (PH) assumption (Table S7).

**E-values**

The E-value represents the minimum strength of association that an unmeasured confounder would need to have with both the treatment and the outcome to fully explain away a specific treatment-outcome association, conditional on the measured covariates. In the Cox models, the E-values are all larger than the magnitude of each adjusted hazard ratio for collagen and neurological AEs when comparing FQ with SXT and FQ with NTF (Table 5). To explain the observed adjusted hazard ratio of 1.25 for tendon rupture, an unmeasured confounder with an adjusted hazard ratio of 1.8 would be needed to render the described association as nonsignificant. A large E-value implies considerable unmeasured confounding would be needed to explain an effect estimate observed. A small E-value implies little unmeasured confounding would be needed to explain an effect estimate observed.

***Duration of index antibiotic treatment***

The mean (SD) treatment duration, added *post hoc*, was 6.44 (2.21) for the FQ cohort with a median duration of 7 days. For NTF and SXT the mean (SD) treatment duration was 6.66 (1.49) and 6.31 (2.43) days respectively, with a median duration of 7 days for both treatments. For AMC the mean (SD) treatment duration was 8.33 (1.92) with a median duration of 10 days (Table S8).

**SUPPLEMENTARY TABLES AND FIGURES**

**TABLE S1** Patient eligibility according to International Classification of Diseases Ninth and Tenth Revision (ICD-9/ICD-10) codes

| **Conditions** | **ICD-9** | **ICD-10 codes** |
| --- | --- | --- |
| UTI | 595.0, 595.9, 599.0 | N30.00, N30.01, N30.90, N30.91, N39.0 |
| *Genetic syndromes or autoimmune diseases were excluded due to potential association with outcomes* | | |
| Marfan Syndrome | 759.82 | Q87.4 |
| Ehlers-Danlos Syndrome | 756.83 | Q79.6 |
| Loeys-Dietz Syndrome | 759.89 | Q87.89 |
| Takayasu’s Disease | 446.7 | M31.4 |
| Biscupid Aortic Valve | 746.4 | Q23.1 |
| Turners Syndrome | 758.6 | Q96 |
| Irritable Bowel Disease | CID 578 | CID 578 |
| Systemic Lupus Erythematosus | 710.0 | M32.9 |
| Systemic Sclerosis | 710.1 | M34.1 |
| Sica Syndrome | 710.2 | M35 |
| Dermatomyositis | 710.3 | M33.10 |
| Hereditary Peripheral Neuropathy | 356.0 | G60.9 |
| *Comorbidities and comedications of interest* | | |
| Myocardial infarction | 410.x; 412.x | I21.x; I22.x; I252 |
| Congestive heart failure | 398.91; 402.01; 402.11; 402.91; 404.01; 404.03; 404.11; 404.13; 404.91; 404.93; 425.4; 425.5; 425.6; 425.7; 425.8; 425.9; 428.x | I09.9; I11.0; I13.0; I13.2; I25.5; I42.0; I42.5; I42.6; I42.7; I42.8; I42.9; I43.x; I50.x; P29.0 |
| Peripheral vascular disease | 440.x; 437.3; 443.1; 443.2; 443.8; 443.9; 447.1; 557.1; 557.9; V43.4 | I70.x; I173.x; I73.1; I73.8; I73.9; I77.1; I79.1; I79.8; K55.1; K55.8; K55.9; Z95.8; Z95.9 |
| Cerebrovascular disease | 430.x; 431.x; 432.x; 433.x; 434.x; 435.x; 436.x; 437.x; 438.x | G45.x; G46.x; H34.0x; H34.1x; I60.x; I61.x; I62.x; I63.x; I64.x; I65.x; I66.x; I67.x; I68.x |
| Dementia | 290.0; 290.1x; 290.2x; 290.3; 290.4x; 294.0; 294.1x; 294.2x; 294.8; 331,0; 331.1x; 331.2; 331.7; 797 | F01.x; F02.x; F03.x; F04; F05; F06.1; F06.8; G13.2; G13.8; G30.x; G31.0x; G31.1; G31.2; G91.4; G94; R41.81; R54 |
| Chronic obstructive pulmonary disease | 490.x; 491.x; 492.x; 493.x; 494.x; 495.x; 496.x; 500.x; 501.x; 502.x; 503.x; 504.x; 505.x; 506.4; 508.1; 508.8 | J40.x; J41.x; J42.x; J43.x; J44.x; J45.x; J46.x; J47.x; J60.x; J61.x; J62.x; J63.x; J64.x; J65.x; J66.x; J67.x; J68.4; J70.1; J70.3 |
| Connective tissue disease/rheumatic disease | 446.5; 710.4; 714.0; 714.1; 714.2; 714.8x; 725.x | M05.x; M06.x; M31.5; M35.1; M35.3; M36.0 |
| Peptic ulcer disease | 531.x; 532.x; 533.x; 534.x | K25.x; K26.x; K27.x; K28.x |
| Liver disease (mild) | 070.22; 070.23; 070.32; 070.33; 070.44; 070.54; 070.6; 070.9; 570.x; 571.x; 573.3; 573.4; 573.8; 573.9; V42.7 | B18.x; K70.0; K70.1; K70.2; K70.3; K70.9; K71.3; K71.4; K71.5; K71.7; K73.x; K74.x; K76.0; K76.2; K76.3; K76.4; K76.8; K76.9; Z94.4 |
| Diabetes without chronic complications | 250.8x; 250.9x; 249.0x; 249.1x; 249.2x; 249.3x; 249.9x | E08; E09; E10; E11; E13 (relevant subcodes: E**.0x; E**.1x; E**.6x; E**.8x; E**.9x) |
| Renal disease (mild to moderate) | 403.00; 403.10; 403.90; 404.00; 404.01; 404.10; 404.11; 404.90; 404.91; 582.x; 583.x; 583.1; 583.2; 585.3; 585.4; 585.9; V42.0 | I12.9; I13.0; I13.10; N03.x; N05.x; N18.1; N18.2; N18.3; N18.4; N18.9; Z94.0 |
| Diabetes mellitus with chronic complications | 250.4; 250.5; 250.6; 250.7 | E08; E09; E10; E11; E13 (relevant subcodes: E**.2; E**.3; E**.4; E**.5) |
| Hemiplegia or Paraplegia | 334.1; 342.x; 434.x; 344.x | G04.1; G11.4; G80.1; G80.2; G81.x; G82.x; G83.x |
| Cancer (includes: leukemia, lymphoma, and other malignancy) | I4x.x; I5x.x; I6x.x; I70.x; I71.x; I72.x; I74.x; I76.x; I79.x; I8x.s; I90.x; I91.x; I 92.x; I93.x; I94.x; I95.x; I99.1; 200.x; 201.x; 202.x; 203.x; 204.x; 205.x; 206.x; 207.x; 208.x; 238.6 | C0x.x; C1x.x; C2x.x; C30.x; C31.x; C32.x; C33.x; C34.x; C37.x; C38.x; C39.x; C40.x; C41.x; C43.x; C45.x; C46.x; C47.x; C48.x; C50; C51-58.x; C60-63.x; C76.x; C80.1; C81.x; C82.x; C83.x; C84.x; C85.x; C88.x; C9x.x |
| Liver disease (moderate or severe) | 456.0; 456.1; 456.2x; 572.2; 572.3; 572.4; 572.8 | I85.0x; I86.4; K70.4x; K71.1x; K72.1x; K72.9; K76.5; K76.6; K76.7 |
| Renal disease (severe) | Excluded therefore not applicable for CCI | Excluded therefore not applicable for CCI |
| Human immunodeficiency virus (HIV) infection, no acquired immunodeficiency syndrome (AIDS) | Excluded therefore not applicable for CCI | Excluded therefore not applicable for CCI |
| Metastatic carcinoma | 196.x; 197.x; 198.x; 199.0 | C77.x; C78.x; C79.x; C80.0; C80.2 |
| HIV/AIDS^†^ | Excluded therefore not applicable for CCI | Excluded therefore not applicable for CCI |
| *Comedications* | | |
| Diabetes medication,  corticosteroids systemic, aminoglycosides, tetracyclines, statins, Isoniazid | Source codes: CID543; CID897; CID928; CID775; HEDIS | - |

*Renal Disease Severe (CCI domain 16). ^†^HIV infection/no AIDS (CCI domain 17), AIDS (CCI domain 19), and Diabetes mellitus with chronic complications (CCI domain 12) are excluded from the study and not evaluated as per the CCI. Other exclusion criteria are removed from the CCI categories (autoimmune diseases, outcomes).

CCI, Charleston Comorbidity Index.

**TABLE S2** Definitions

| **Terminology** | **Explanation** |
| --- | --- |
| Crude absolute risk | The cumulative probability of an event (*N* of events per group/*N* people in that group) and the cumulative hazard rate measured through KM curves.  Usually expressed as % and not a comparison between 2 treatments. |
| Adjusted absolute risk | The cumulative probability of an event after controlling for baseline differences (*N* events per group/*N* people in that group) and the cumulative hazard rate measured through KM curves. Expressed as a %. |
| Crude hazard ratio | The ratio of the probability of an event (AESI) occurring with exposure (antibiotic treatment) relative to the probability of the event (AESIs) occurring without exposure (antibiotic treatment) over a unit of time. The hazard ratio is an effect size measure for time-to-event data. Exposure relates to FQ versus. SXT or NFT or AMC. |
| Adjusted hazard ratio | Hazard ratio after adjusting for differences in patient demographic and clinical characteristics across the groups being compared (e.g. FQ treated uUTI versus NTF or SXT treated uUTI). |

AESIs, adverse events of special interest; AMC, amoxicillin clavulanate; FQ, fluroquinolone; KM, Kaplan Meier; NTF, nitrofurantoin; SXT, trimethoprim-sulfamethoxazole; uUTI, uncomplicated urinary tract infection.

**TABLE S3** Baseline patient characteristics per index antibiotic treatment (FQ versus AMC)

|  | **Index exposure groups** | | |
| --- | --- | --- | --- |
| **Baseline characteristics** | **Overall**  **(*N* = 954,777)** | **FQ**  **(*N* = 386,537)** | **AMC**  **(*N* = 16,535)** |
| Mean age at index (years±SD) | 50.3±20.1 | 54.0±19.3 | 54.5±21.6 |
| **Race**, n (%) |  |  |  |
| White | 651,557 (68.2) | 261,108 (67.6) | 10,528 (63.7) |
| Black | 88,895 (9.3) | 36,386 (9.4) | 1,613 (9.8) |
| Asian | 34,897 (3.7) | 14,614 (3.8) | 592 (3.6) |
| Hispanic | 115,822 (12.1) | 49,234 (12.7) | 2,493 (15.1) |
| Unknown | 63,606 (6.7) | 25,195 (6.5) | 1,309 (7.9) |
| **Treatment in prior 12 months**, n (%) |  |  |  |
| Antimicrobial exposure* | 496,382 (52.0) | 207,138 (53.6) | 9,771 (59.1) |
| All-hospitalization | 49,535 (5.2) | 22,830 (5.9) | 1,570 (9.5) |
| Physician visits | 944,531 (98.9) | 382,281 (98.9) | 16,187 (97.9) |
| ≥1 UTI episode | 154,969 (16.2) | 67,644 (17.5) | 3,962 (24.0) |
| **Recurrent uUTI**, n (%) |  |  |  |
| 1 episode in prior 6 months or 2 in prior 12 | 67,270 (7.0) | 29,507 (7.6) | 1,929 (11.7) |
| 1 episode in prior 6 months | 58,949 (6.2) | 25,829 (6.7) | 1,701 (10.3) |
| 2 episodes in prior 12 months | 27,402 (2.9) | 12,103 (3.1) | 897 (5.4) |
| **Drug use in prior 90 days**, n (%) |  |  |  |
| Diabetes drug(s) | 48,232 (5.1) | 21,860 (5.7) | 1,110 (6.7) |
| Corticosteroids | 49,289 (5.2) | 21,160 (5.5) | 1,513 (9.2) |
| Aminoglycosides | 18 (0.0) | 7 (0.0) | 0 |
| Tetracyclines | 8,220 (0.9) | 3,086 (0.8) | 192 (1.2) |
| Statins | 141,257 (14.8) | 69,087 (17.9) | 2,897 (17.5) |
| Gabapentinoids | 4,408 (0.5) | 2,004 (0.5) | 110 (0.7) |
| Isoniazid | 0 | 0 | 0 |
| **Comorbidities**, n (%) |  |  |  |
| Total | 954,777 (100.0) | 386,537 (100.0) | 16,535 (100.0) |
| Myocardial Infarction | 754 (<1) | 384 (<1) | 23 (<1) |
| Congestive Heart Failure | 2,273 (<1) | 1,205 (<1) | 107 (<1) |
| Peripheral Vascular Disease | 2,286 (<1) | 1,254 (<1) | 82 (<1) |
| Cerebrocardiovascular Disease | 1,918 (<1) | 961 (<1) | 54 (<1) |
| Dementia | 3,610 (<1) | 1,893 (<1) | 85 (<1) |
| Chronic obstructive pulmonary disease | 13,376 (1.4) | 6,754 (1.7) | 639 (3.9) |
| Connective Tissue/Rheumatic Disease | 1,060 (<1) | 585 (<1) | 32 (<1) |
| Peptic Ulcer Disease | 281 (<1) | 150 (<1) | 6 (<1) |
| Mild Liver Disease | 1,877 (<1) | 1,010 (<1) | 52 (<1) |
| Diabetes without complication | 12,308 (1.3) | 5,592 (1.4) | 422 (2.6) |
| Renal Disease Mild to Moderate | 938 (<1) | 566 (<1) | 32 (<1) |
| Paraplegia and Hemiplegia | 165 (<1) | 81 (<1) | 5 (<1) |
| Cancer | 3,028 (<1) | 1,662 (<1) | 81 (<1) |
| Severe Liver Disease | 59 (<1) | 29 (<1) | 6 (<1) |
| Metastatic Carcinoma | 244 (<1) | 155 (<1) | 5 (<1) |

*Antimicrobial exposure 91–364 days prior to the index date. Patient data was obtained from Optum’s de-identified Clinformatics Data Mart Database.
AMC, amoxicillin clavulanate; FQ, fluoroquinolone; SD, standard deviation; UTI, urinary tract infection.

**TABLE S4** Descriptive number (%) of collagen and neurological AESIs (without the application of Kaplan-Meier method) by index antibiotic treatment

|  | **Index antibiotic treatment** | |
| --- | --- | --- |
| **Collagen and neurological AESIs** | **FQ** (*N* = 386,537) | **AMC** (*N* = 16,535) |
| **Collagen AESIs**, n (%) |  |  |
| Total | 1,200 (0.31) | 73 (0.44) |
| Tendon rupture | 308 (0.08) | 18 (0.11) |
| Retinal detachment | 173 (0.05) | 13 (0.08) |
| Uveitis | 262 (0.07) | 13 (0.08) |
| Aortic aneurysm | 444 (0.11) | 27 (0.16) |
| Aortic dissection | 25 (0.01) | 2 (0.01) |
| **Neurological AESIs**, n (%)* |  |  |
| Total | 18,899 (4.89) | 1,000 (6.05) |
| CNS | 3,722 (0.96) | 254 (1.54) |
| PNS | 16,420 (4.25) | 842 (5.09) |

*Some patients experienced both CNS and PNS AESIs.

AESIs, adverse events of special interest; AMC, amoxicillin clavulanate; CNS, central nervous system; FQ, fluoroquinolone; PNS, peripheral nervous system.

**TABLE S5** Crude absolute risk of collagen and neurological AESIs by index antibiotic treatment (with censoring)

| **Collagen and neurological AESIs** | **Index antibiotic treatment**  **Crude absolute risk, % (95% CI)*** | |
| --- | --- | --- |
|  | **FQ** | **AMC** |
| Any collagen AESIs | 0.31 (0.29–0.33) | 0.39 (0.30–0.51) |
| Tendon rupture | 0.08 (0.07–0.09) | 0.08 (0.04–0.14) |
| Retinal detachment | 0.05 (0.04–0.05) | 0.08 (0.05–0.15) |
| Uveitis | 0.07 (0.06–0.08) | 0.08 (0.04–0.14) |
| Aortic aneurysm | 0.11 (0.10–0.12) | 0.14 (0.09–0.22) |
| Aortic dissection | 0.01 (0.00–0.01) | 0.02 (0.00–0.06) |
| Any neurological AESIs | 4.76 (4.69–4.84) | 5.90 (5.51–6.31) |
| CNS | 0.90 (0.87–0.93) | 1.43 (1.24–1.64) |
| PNS | 4.14 (4.07–4.21) | 4.94 (4.58–5.32) |

*Assessed from Day 1–90.

AESIs, adverse events of special interest; AMC, amoxicillin clavulanate; CI, confidence interval; CNS, central nervous system; FQ, fluoroquinolone; PNS, peripheral nervous system.

**TABLE S6** Adjusted absolute risk of collagen and neurological adverse events by index antibiotic treatment (after sIPTW)

| **Collagen and neurological AESIs** | **Index antibiotic treatment**  **Adjusted absolute risk, % (95% CI)*** | |
| --- | --- | --- |
|  | **FQ** | **AMC** |
| Any collagen AESIs | 0.31 (0.29–0.33) | 0.36 (0.26–0.49) |
| Tendon rupture | 0.08 (0.07–0.09) | 0.10 (0.05–0.18) |
| Retinal detachment | 0.05 (0.04–0.05) | 0.09 (0.05–0.17) |
| Uveitis | 0.07 (0.06–0.08) | 0.04 (0.02–0.11) |
| Aortic aneurysm | 0.11 (0.10–0.12) | 0.12 (0.07–0.21) |
| Aortic dissection | 0.01 (0.00–0.01) | 0.01 (0.00–0.08) |
| Any neurological AESIs | 4.78 (4.70–4.85) | 5.69 (5.25–6.16) |
| CNS | 0.90 (0.87–0.94) | 1.29 (1.09–1.53) |
| PNS | 4.15 (4.09–4.22) | 4.78 (4.37–5.22) |

*Absolute risk (%) after sIPTW for: age, race/ethnicity, region, year of index, uUTI recurrence, prior antimicrobial exposure, prior hospitalization, prior physician visits, prior UTI, comorbidities, and comedications. FQ group based on propensity scores generated for comparison with AMC. Assessed from Day 1–90 (before censoring).

Collagen AESIs comprised tendon rupture, aortic aneurysm with/without dissection, retinal detachment, uveitis and a composite category for all collagen AESIs. Neurological AESIs comprised CNS AESIs (seizures/convulsions, intracranial hypertension, psychosis/delirium and altered mental status/encephalopathy), PNS AESIs (muscle weakness, paresthesia/sensory disturbance [tingling, numbness, burning pain, allodynia], gait dysfunction, peripheral neuropathy) and a composite category for all CNS and PNS AESIs.

AESIs, adverse events of special interest; AMC, amoxicillin clavulanate; CI, confidence interval; CNS, central nervous system; FQ, fluoroquinolone; PNS, peripheral nervous system; sIPTW, inverse probability of treatment weighting; uUTI, uncomplicated urinary tract infection.

**TABLE S7** Adjusted hazard ratio of collagen and neurological AESIs by index antibiotic treatment (after sIPTW)

| **AESIs** | **Adjusted hazard ratio (95% CI)** | ***P*-Value** | **E-Value** | **PH Assumption**  ***P*-value*** |
| --- | --- | --- | --- | --- |
| **Collagen AESIs: FQ versus AMC** | | | | |
| Any collagen AE | 0.86 (0.65–1.16) | 0.3247 | 1.5847 | 0.9892 |
| Tendon rupture | 0.79 (0.45–1.38) | 0.4157 | 1.8323 | 0.5805 |
| Retinal detachment | 0.51 (0.28–0.92) | 0.0257 | 3.3443 | 0.7763 |
| Uveitis | 1.64 (0.71–3.79) | 0.2477 | 2.6634 | 0.5789 |
| Aortic aneurysm | 0.94 (0.57–1.55) | 0.8046 | 1.3294 | 0.6014 |
| Aortic dissection | 0.76 (0.11–5.09) | 0.7810 | 1.9442 | 0.5658 |
| **Neurological AESIs: FQ versus AMC** | | | | |
| Any neurological AE | 0.84 (0.78–0.90) | <0.0001 | 1.6715 | 0.4955 |
| CNS | 0.70 (0.60–0.81) | <0.0001 | 2.2191 | 0.7443 |
| PNS | 0.87 (0.80–0.94) | 0.0007 | 1.5602 | 0.3004 |

*Assessed from Day 1–90. *P* < 0.05 is considered as the violation of PH assumption.

AESIs, adverse events of special interest; AMC, amoxicillin clavulanate; CI, confidence interval; CNS, central nervous system; FQ, fluoroquinolone; PH, proportional hazards; PNS, peripheral nervous system; sIPTW, inverse probability of treatment weighting.

**Table S8** Mean and median treatment duration of index antibiotic treatment

| **Index antibiotic treatment** | **Number of patients** | **Mean duration, days (SD)** | **Median duration, days (range: min, max)** |
| --- | --- | --- | --- |
| FQ | 386,537 | 6.44 (2.21) | 7 (3, 10) |
| NTF | 314,585 | 6.66 (1.49) | 7 (3, 10) |
| SXT | 237,120 | 6.31 (2.43) | 7 (3, 10) |
| AMC | 16,535 | 8.33 (1.92) | 10 (3, 10) |
| Overall | 954,777 | 6.52 (2.08) | 7 (3, 10) |

AMC, amoxicillin-clavulanic acid; FQ, fluoroquinolone; NTF, Nitrofurantoin; SD, standard deviation; SXT, trimethoprim/sulfamethoxazole.

**FIG S1** Crude and adjusted absolute risk (%) over time of (**A** and **B**) collagen AESIs and (**C** and **D**) neurological AESIs with FQ versus AMC


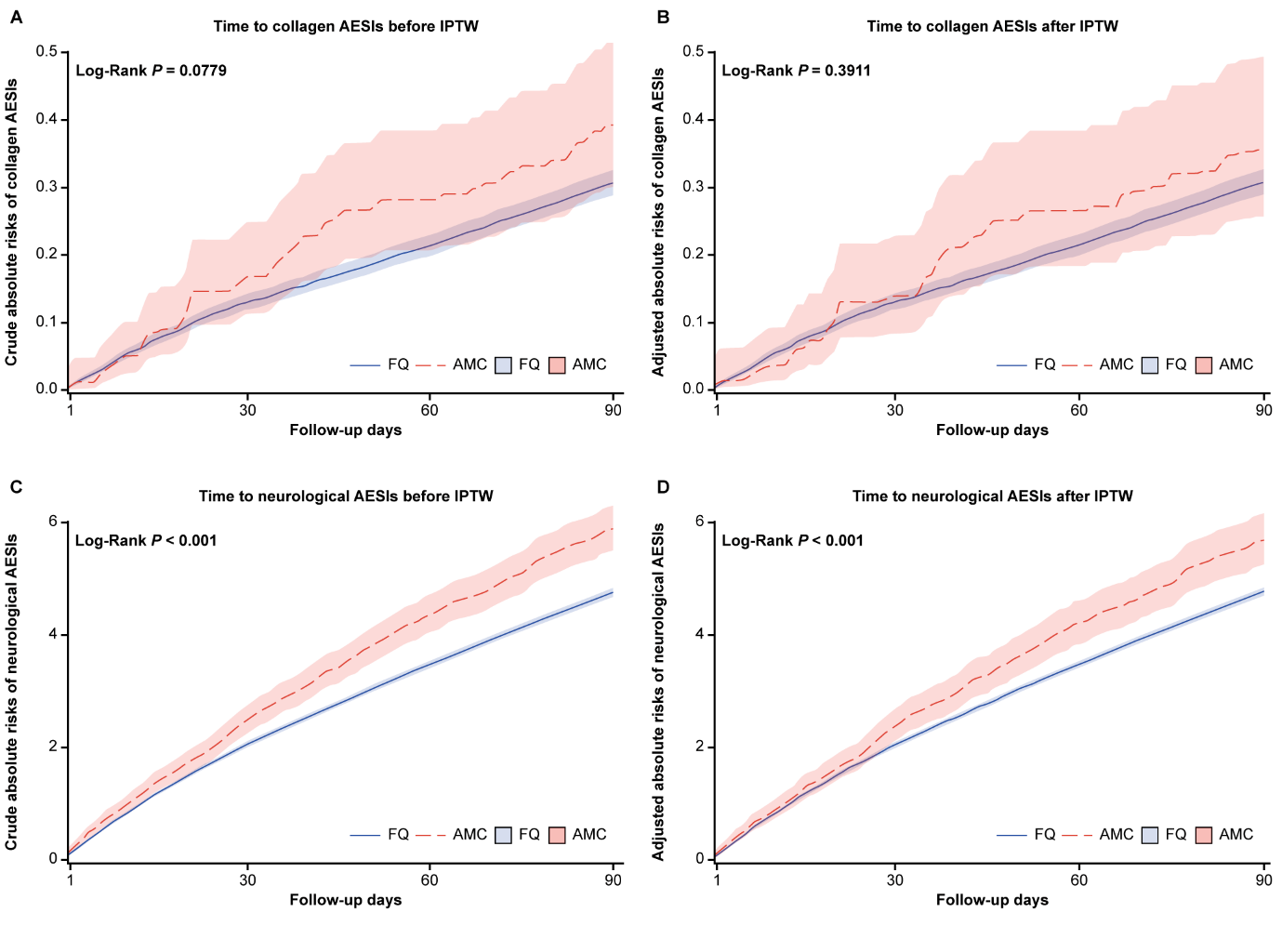


Collagen AESIs comprised tendon rupture, aortic aneurysm with or without dissection, retinal detachment, uveitis, and a composite category for all collagen AESIs. Neurological AESIs comprised CNS AESIs (seizures/convulsions, intracranial hypertension, psychosis/delirium, and altered mental status/encephalopathy), PNS AESIs (muscle weakness, paresthesia/sensory disturbance [tingling, numbness, burning pain, allodynia], gait dysfunction, peripheral neuropathy), and a composite category for all CNS and PNS AESIs.

AESIs, adverse events of special interest; AMC, amoxicillin clavulanate; FQ, fluoroquinolone; sIPTW, inverse probability of treatment weighting.

**FIG S2** Forest plot of crude and adjusted absolute risk of (**A**) collagen, and (**B**) neurological AESIs in FQ- versus AMC-treated patients


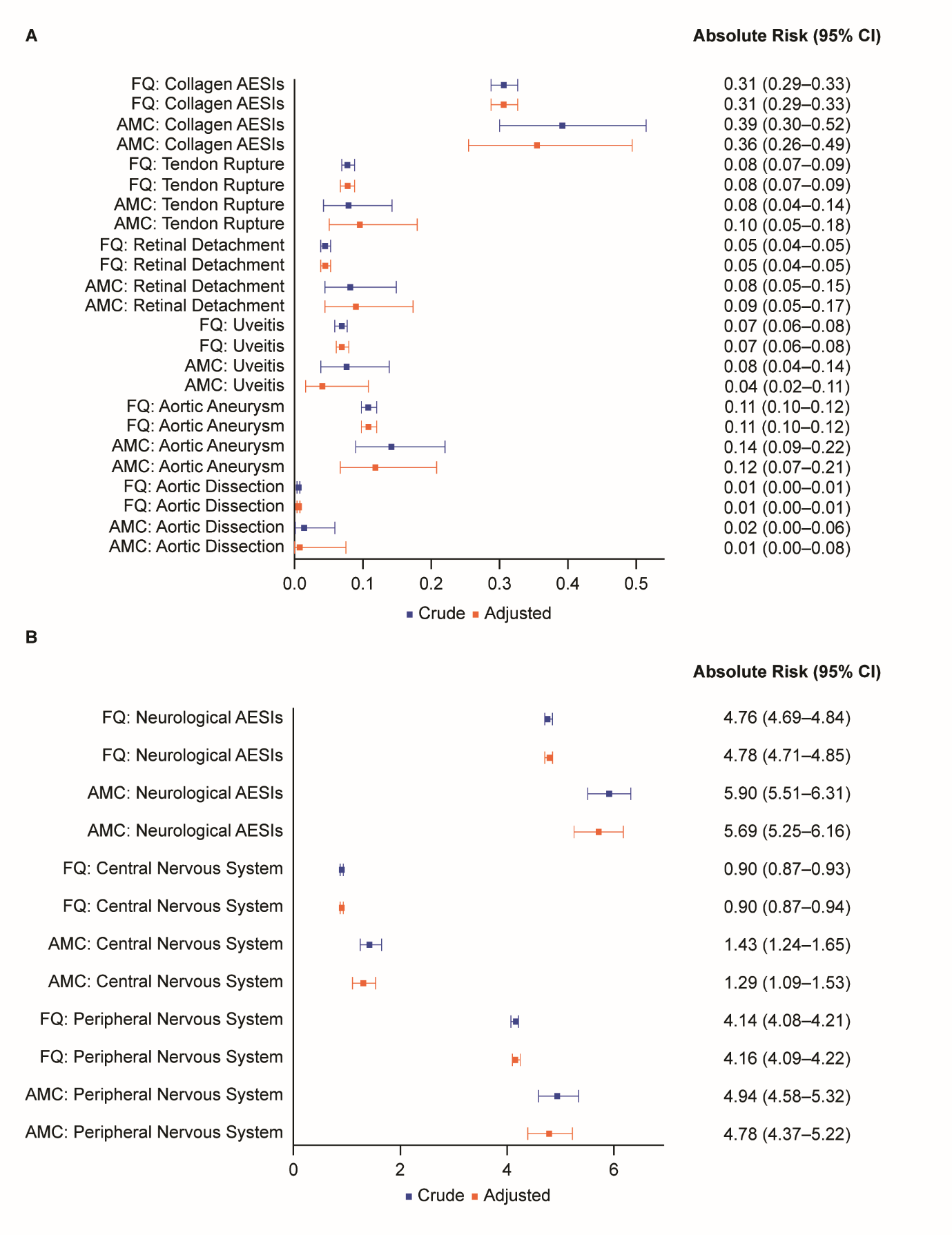


Collagen AESIs comprised tendon rupture, aortic aneurysm with or without dissection, retinal detachment, uveitis, and a composite category for all collagen AESIs. Neurological AESIs comprised CNS AESIs (seizures/convulsions, intracranial hypertension, psychosis/delirium, and altered mental status/encephalopathy), PNS AESIs (muscle weakness, paresthesia/sensory disturbance [tingling, numbness, burning pain, allodynia], gait dysfunction, peripheral neuropathy), and a composite category for all CNS and PNS AESIs.

AESIs, adverse events of special interest; AMC, amoxicillin clavulanate; CI, confidence interval; CNS, central nervous system; FQ, fluoroquinolone; HR, hazard ratio; NTF, nitrofurantoin; PNS, peripheral nervous system; sIPTW, inverse probability of treatment weighting; UTI, urinary tract infection; uUTI, uncomplicated urinary tract infection.

**FIG S3** Forest plot of crude and adjusted hazard ratio for (**A**) collagen, and (**B**) neurological AESIs in FQ- versus AMC-treated patients


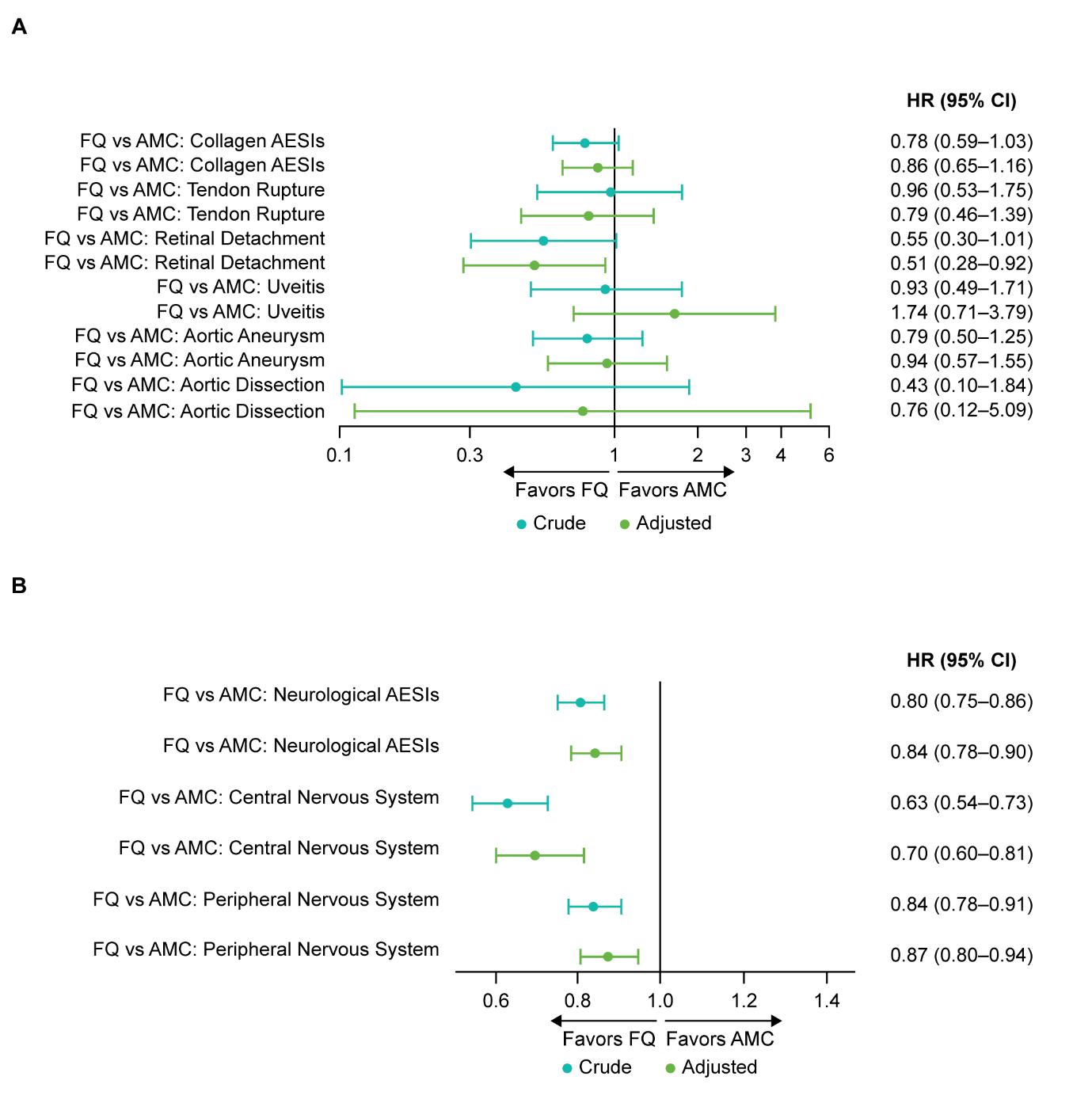


Collagen AESIs comprised tendon rupture, aortic aneurysm with or without dissection, retinal detachment, uveitis, and a composite category for all collagen AESIs. Neurological AESIs comprised CNS AESIs (seizures/convulsions, intracranial hypertension, psychosis/delirium, and altered mental status/encephalopathy), PNS AESIs (muscle weakness, paresthesia/sensory disturbance [tingling, numbness, burning pain, allodynia], gait dysfunction, peripheral neuropathy), and a composite category for all CNS and PNS AESIs. HRs are adjusted for: age, race/ethnicity, region, year of new uUTI, uUTI recurrence, prior antimicrobial exposure, prior hospitalization, prior physician visits, prior UTI, comorbidities, and comedications.

AESIs, adverse events of special interest; AMC, amoxicillin clavulanate; CI, confidence interval; CNS, central nervous system; FQ, fluoroquinolone; HR, hazard ratio; NTF, nitrofurantoin; PNS, peripheral nervous system; sIPTW, inverse probability of treatment weighting; UTI, urinary tract infection; uUTI, uncomplicated urinary tract infection.
